# Atypical imaging observations of branchial cleft cysts

**DOI:** 10.3892/ol.2013.1656

**Published:** 2013-11-04

**Authors:** SU HU, CHUN-HONG HU, LING YANG, JIAN-MING XING, JIAN-HUA CHEN, ZI-LI GE, JI-SHENG LIU

**Affiliations:** 1 Department of Radiology, The First Affiliated Hospital of Soochow University, Suzhou, Jiangsu 215006, P.R. China; 2 Department of Oral Surgery, The First Affiliated Hospital of Soochow University, Suzhou, Jiangsu 215006, P.R. China; 3 Department of Otorhinolaryngology, The First Affiliated Hospital of Soochow University, Suzhou, Jiangsu 215006, P.R. China

**Keywords:** neck, branchial cleft cysts, computerized tomography, magnetic resonance imaging

## Abstract

The aim of the present study was to assess the atypical imaging manifestations of branchial cleft cysts (BCCs) confirmed by pathology. Computerized tomography (CT) or magnetic resonance imaging (MRI) of 17 BCC cases were reviewed. The imaging features, including laterality, location, border, attenuation and internal architecture, were evaluated. All 17 cases were second BCCs, including 5 cases of Bailey type I classification cysts and 12 cases of type II classification cysts. The atypical imaging features included signal and morphological abnormalities. The abnormal signal intensities were caused by intracapsular bleeding (n=2) or solidification of cystic fluid (n=2). Intracystic hemorrhaging revealed homogeneous hyperintensity on T1-weighted image (T1WI) and T2-weighted image (T2WI). Solidification of cystic fluid revealed slightly homogeneous hyperintensity compared with muscle on T1WI and homogeneous hypointensity on T2WI without enhancement. The aberrant morphology mainly presented as thickening of the cystic wall (n=13). Thickened walls of BCCs with ill- (n=5) or well- (n=8) defined borders were observed in 13 patients. In 3 patients, significant enhancement was identified following intravenous gadolinium administration (n=4). When with atypical CT or MRI features are presented, the typical location of BCCs can help in the diagnosis, as it is located at the lateral portion of the neck adjacent to the anterior border of the mandibular angle or sternocleidomastoid muscle. The atypical observations, including variable signals, imply that the cystic content has changed. Thickened walls indicate inflammation or cancerous tendency and patients with ill-defined margins, vascular involvement or lymphadenopathy atelectasis indicate malignant conversion.

## Introduction

Branchial cleft cysts (BCCs) are the most common congenital masses of the lateral neck and are caused by abnormal embryonic development (1). Second BCCs are the most common subtype of BCCs and are responsible for ~95% of all cases (2). Second BCCs are divided into four types based on Bailey’s criterion (3). In previous studies, typical imaging observations of BCCs have been described (4). However, the imaging appearances of BCCs may be atypical under specific pathological conditions, including secondary infection, hemorrhaging or malignant transformation. The present study retrospectively evaluated computerized tomography (CT) or magnetic resonance imaging (MRI) observations of pathologically confirmed BCCs.

## Materials and methods

### Patients

In total, 17 patients (11 male and 6 female; age range, 15–88 years; mean age, 39.1 years) with BCCs were reviewed retrospectively. In these patients, infection, hemorrhaging or malignant transformation of BCCs was confirmed by surgical or pathological analysis. In total, 7 of the 17 patients underwent CT examinations, and MRI scans were performed on an additional 10 patients. All patients exhibited painless swelling, however, 10 patients had a past history of repeated pain and an increase in the size of the swelling which had been resolved with antibiotics. The duration of symptoms varied between 10 days and 14 years. The study was approved by the ethics committee of the First Affiliated Hospital of Soochow University. Wriitten informed content was obtained from the patients.

### CT examination

CT scans were performed on a 64-row MDCT scanner (Somaton Sensation 64; Siemens Healthcare, Erlangen, Germany) with a beam pitch of 0.8, section thickness of 5.0 mm and reconstruction increment of 4.7 mm.

### MRI examination

MRI examination was performed on a 0.5 T MRI unit (GE Vectra; GE Healthcare, Amersham, UK; n=3) and 1.5 T MRI unfit (Philips Eclipse; Philips Healthcare, Amsterdam, The Netherlands; n=7). The protocol for MRI for all patients was as follows: i) axial spin echo (SE) T1-weighted imaging (T1WI; repetition time (TR)/echo time(TE), 400–500/12–30 ms; n=10); ii) axial fast SE (FSE) T2-weighted imaging (T2WI; TR/TE, 3,800–4,500/100–112 ms; n=10); iii) coronal FSE T2WI (TR/TE, 3,500/112 ms; n=10); and iv) time of flight MR angiography (MRA; TR/TE, 27/6.7 ms; n=4). Postcontrast T1WI was performed following administration of 0.1–0.2 mmol/kg body weight gadolinium (Gd-DTPA; n=6).

### Imaging analysis

All images were interpreted retrospectively by the consensus of two radiologists with four- and three-years experience, respectively, in head and neck imaging. The following characteristics of each lesion were analyzed: Laterality, location, border, attenuation or signal intensity and internal architecture.

## Results

### Patient diagnosis

All 17 cases exhibited with second BCCs with 12 cases located on the left and 5 cases on the right. According to the Bailey’s criterion, there were 5 cases of type I and 12 cases of type II.

### Atypical imaging features

The atypical imaging features included signal and morphological abnormalities. The abnormal signal intensities were caused by intracapsular bleeding (n=2) or solidification of cystic fluid (n=2). Aberrant morphology mainly presented as the thickening of the cystic wall (n=13).

### Pathological analysis

**Figure 1:**
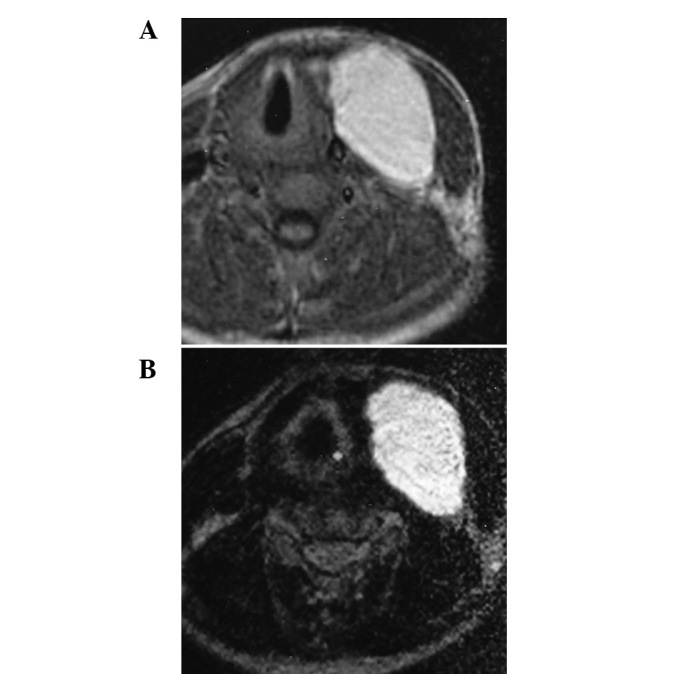
Type II BCC with intracystic hemorrhaging. A left, well-circumscribed mass appeared hyperintense on (A) T1WI and (B) T2WI of the neck. BCC, branchial cleft cysts; T1WI, T1-weighted image; T2WI, T2-weighted image.

**Figure 2:**
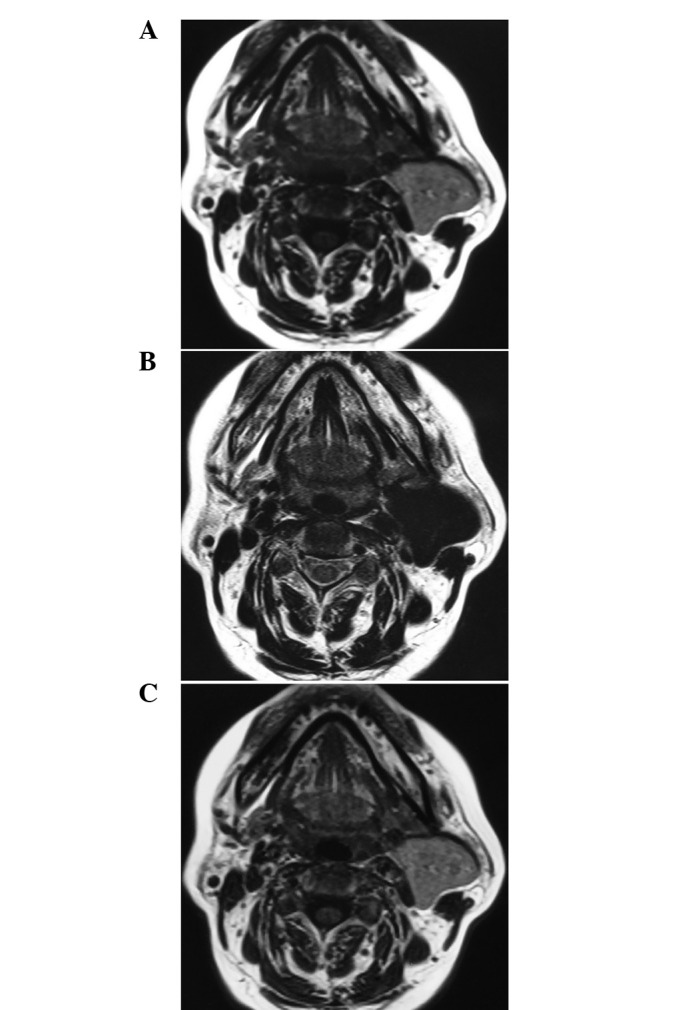
Type I BCC with solidification of cystic fluid. A left side, well-circumscribed mass appeared (A) mild hyperintense similar to the muscle on T1WI and (B) hypointense on T2WI. (C) Following the administration of contrast materials, no significant enhancement was observed. BCC, branchial cleft cyst; T1WI, T1-weighted image; T2WI, T2-weighted image.

Intracystic hemorrhaging was observed in 2 cases. Hemorrhaging appeared as homogeneously hyperintense on T1WI and T2WI (Fig. 1). In gross pathology, the content of the cysts was a dark-red liquid and the solidification of cystic fluid was confirmed in an additional 2 cases, which appeared as jelly-like contents. Solidification of cystic fluid showed slightly homogeneous hyperintensity compared with muscle on T1WI and was homogeneously hypointensitive on T2WI. Following the administration of Gd-DTPA, no significant enhancement was observed (Fig. 2). Keratinizing and non-keratinizing epithelial cells were observed pathologically.

**Figure 3:**
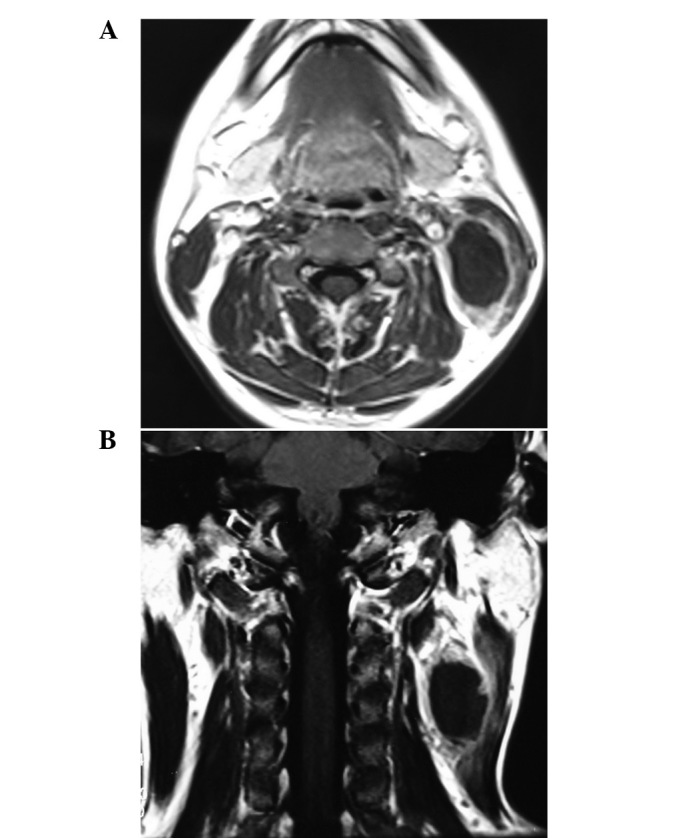
Type II BCC with infection. (A) A left, ill-defined cystic mass presented with enhanced irregular thickening of the wall on contrast-enhanced T1WI. (B) A small protruding lumen was observed at the lateral wall in coronal planes. BCC, branchial cleft cyst; T1WI, T1-weighted image.

**Figure 4:**
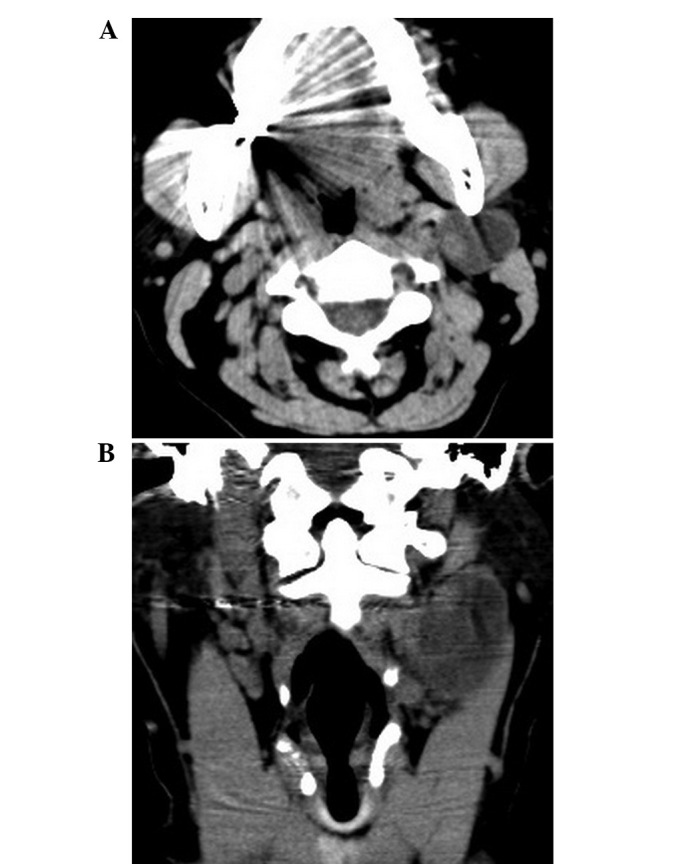
Type II BCC with malignant transformation. (A) A well-defined cystic mass with an intralumenal node was observed on CT scan. (B) In coronal planes, a node located in the inner wall of BCC was observed. BCC, branchial cleft cyst; CT, computerized tomography.

**Figure 5:**
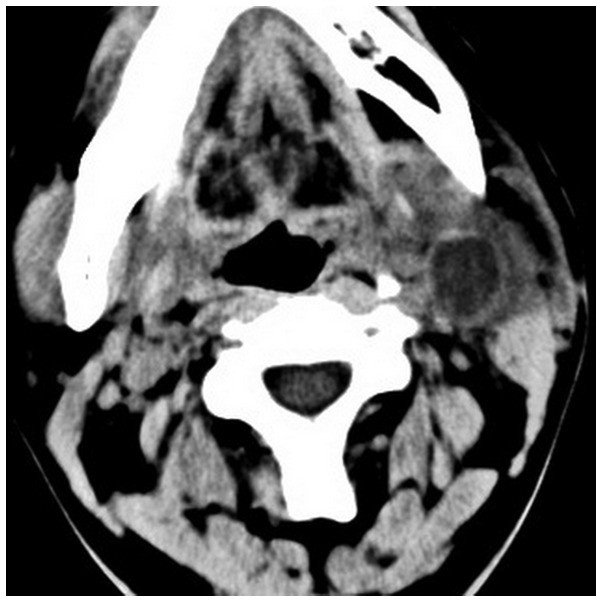
Type II BCC with infection. Axial plain CT scan revealed a left, poorly circumscribed mass with thickened walls in the neck. The density of the sternomastoid muscle and submandibular gland was found to be decreased and the adjacent fat planes were obscured. BCC, branchial cleft cyst; CT, computerized tomography.

**Figure 6:**
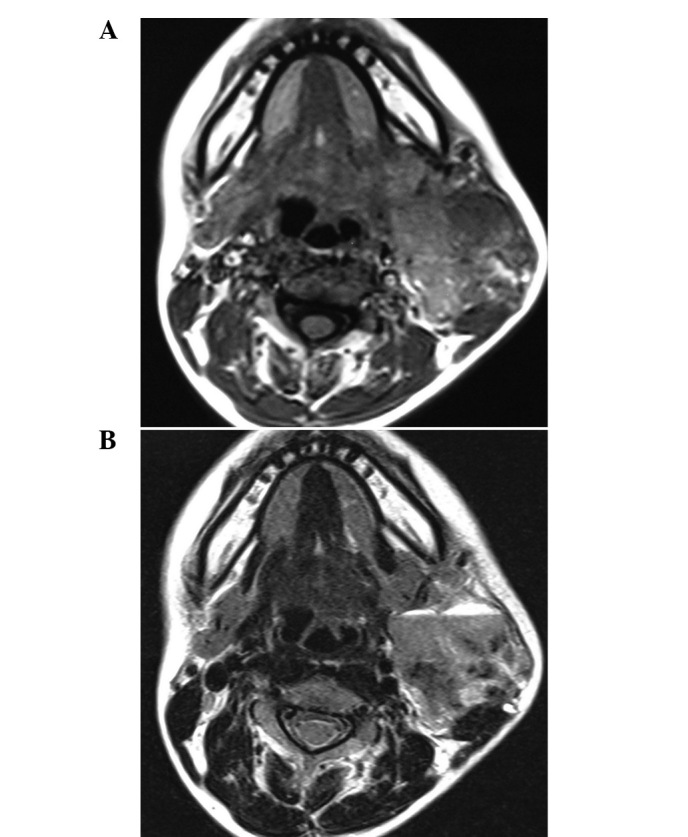
Type II BCC with repeated infection. (A) A heterogeneously isointense and hyperintense mass with an irregular border presented in the left side on T1WI. (B) The cystic section of the mass was narrowed and markedly hyperintense on T2WI. BCC, branchial cleft cyst; T1WI, T1-weighted image; T2WI, T2-weighted image.

Furthermore, thickened cystic walls were observed on CT (n=7) and MRI (n=6). Pathologically, there were 10 cases of infection and 3 cases of carcinomatous transformation. In 4 patients, uniformly-thickened cystic walls with significant enhancement were observed (Fig. 3). Intramural nodes were observed in 3 patients (infection, n=1; malignant transformation, n=2; Figs. 3 and 4) and ill-defined borders were present in 5 patients (infection, n=3; malignant transformation, n=2; Fig. 5). During the surgery, edema of adjacent structures was observed in 3 patients with infection and infiltration of adjacent structures observed in 2 patients with malignant transformation. In one patient, with a recurrent infection for 5 years, the lesion appeared as a solid mass and a small cyst was identified in the lesion. A heterogeneous signal intensity was observed on MRI (Fig. 6). In an additional case with malignant transformation, a lobulated cystic mass with intratumoral septa was observed. The carotid artery sheath was infiltrated and cervical metastasis of the lymph nodes was observed in front of the ipsilateral trapezius.

## Discussion

Generally, the clinical symptoms and imaging observations of BBCs are typical. The majority of BCCs occur between the ages of 10 and 40 years, without gender predilection (1,5). The Bailey classification divides second BCCs into the following four types (3): i) I, the most superficial subtype, which reaches as deep as the platysma surface and lies along the anterior surface of the sternomastoid muscle; ii) II, the most common subtype, identified along the surface of the sternomastoid muscle and posterior to the submandibular gland; iii) III, extends medially at the bifurcation of the internal and external carotid arteries to the lateral pharyngeal wall; and iv) IV, arises in the pharyngeal mucosal space. BCCs often appear as painless masses, which may enlarge and become painful or tender if secondarily infected (1,6,7).

The common imaging observations of BCCs have been described in specific previous studies (1,4,6,8–12). Typical BCCs appear as well-circumscribed, fluid-like masses with uniformly thin walls. The cystic wall shows mild enhancement following the injection of contrast materials. The atypical imaging features are likely to be observed when the lesions are accompanied with infection, hemorrhaging or carcinomatous transformation, which makes diagnosis difficult (1,12).

Intracystic hemorrhaging is usually caused by secondary infection or biopsy attempts. Hemorrhages appears hyperdense on CT scan and shows different signals in the various phases on MRI (11). In the present study, two cases showed high signal intensity on T1WI and T2WI, which must be distinguished from neck lipomas, which also exhibit hyperintensity on T1WI and T2WI. Furthermore, fat-suppression sequences highlight crucial information for determining the diagnosis.

Solidification of cystic fluid is also caused by infection. The cystic fluid becomes muddy due to a rich protein content, which increases the density of BCCs on CT scan and reduces T1 relaxation time (4,11). When the cyst fluid is gradually absorbed, a jelly-like contents is observed which appears hypointense on T2WI, similar to jugular venous aneurysm. Contrast-enhanced MRI and MRA examination is used to differentiate solidification of cystic fluid from jugular venous aneurysm.

The thickening of the cystic wall is often induced by infections (7,13,14). When the wall is markedly thickened following repeated infection, the lesion is likely to appear as a solid mass with a small area of cyst (9). In one case in the present study, the cystic mass was filled with inflammatory tissue. Under these conditions, it is difficult to differentiate BCCs from other solid or cystic tumors in the neck based only on the morphology, density or signal intensity.

BCCs with malignant transformation are rarely observed and its etiology remains unconfirmed. Specific factors may be associated with the malignant transformation of BCCs, including infection, repeated surgery or biopsy and other carcinogenic stimuli (15–17). It is difficult to identify malignancies based on clinical symptoms, imaging observations or even biopsy. The definitive diagnosis relies on pathological examinations following surgical resection (17). In the early stages, only thickened cyst walls with well-defined borders are observed, which are often misinterpreted as infected BCCs, but are unresponsive to antibiotic therapy. One case in the present study was initially considered to be BCCs with infection, however, malignant transformation was confirmed by pathology. The lesion is likely to infiltrate surrounding structures and cause cervical lymphadenopathy atelectasis and distant (lung, bone, liver) metastases in later stages (15,18).

It is difficult to diagnose BCCs preoperatively when the imaging observations are atypical. However, the classical location of the lesion, for example near to the mandibular angle or the anterior sternocleidomastoid muscle, may be indicative (1,19). Signal alternations correlate with the component of cystic contents and the thickened walls of BCCs indicate inflammation or malignant transformation. Malignant transformation must be considered when BCCs exhibit ill-defined borders with adjacent infiltrating vessels, or cause lymphadenopathy atelectasis. Furthermore, it is important to analyze the relative past history of the patient.

## Abbreviations

BCC: branchial cleft cyst
Gd-DTPA: gadolinium
MRI: magnetic resonance image
SE: spin echo
TR: repetition time
TE: echo time
FSE: fast spin echo
MRA: MR angiography
CT: computerized tomography

## References

[1] Koeller KK, Alamo L, Adair CF, Smirniotopoulos JG (1999). Congential cystic masses of the neck: radiologic-pathologic correlation. Radiographics.

[2] Ghosh SK, Kr T, Datta S, Banka A (2006). Parapharyngeal second branchial cyst: A case report. Indian J Otolaryngol Head Neck Surg.

[3] Bailey H (1933). The clinical aspects of branchial cysts. Br J Surg.

[4] Shin JH, Lee HK, Kim SY (2001). Parapharyngeal second brachial cyst manifesting as cranial nerve palsies: MR findings. AJNR Am J Neuroradiol.

[5] Daoud FS (2005). Branchial cyst: an often forgotten diagnosis. Asian J Surg.

[6] Ahuja AT, King AD, Metreweli C (2000). Second branchial cleft cysts: variability of sonographic appearances in adult cases. AJNR Am J Neuroradiol.

[7] Lo Re V, Brennan PJ, Wadlin J (2001). Infected branchial cleft cyst due to *Bordetella bronchiseptica* in an immunocompetent patient. J Clin Microbiol.

[8] Faerber EN, Swartz JD (1991). Imaging of neck masses in infants and children. Crit Rev Diagn Imaging.

[9] Sarioglu S, Unlu M, Adali Y (2012). Branchial cleft cyst with xanthogranulomatous inflammation. Head Neck Pathol.

[10] Saussez S, De Maesschalk T, Mahillon V (2009). Second branchial cyst in the parapharyngealspace: a case report. Auris Nasus Larynx.

[11] Cerezal L, Morales C, Abascal F (1998). Pharyngeal branchial cyst: magnetic resonance findings. Eur J Radiol.

[12] Setoguchi T, Hasuo K, Shida Y (2007). Second branchial cleft cyst with ‘cyst-within-cyst’ appearance. Clin Imaging.

[13] Ingoldby CJ (1985). Unusual presentations of branchial cysts: a trap for the unwary. Ann R Coll Surg Engl.

[14] Ibrahim M, Hammoud K, Maheshwari M, Pandya A (2011). Congenital cystic lesions of the head and neck. Neuroimaging Clin N Am.

[15] Bernstein A, Scardino PT, Tomaszewki MM, Cohen MH (1976). Carcinoma arising in a branchial cleft cyst. Cancer.

[16] Cho JS, Shin SH, Kim HK (2011). Primary papillary carcinoma originated from a branchial cleft cyst. J Korean Surg Soc.

[17] Girvigian MR, Rechdouni AK, Zeger GD (2004). Squamous cell carcinoma arising in a second branchial cleft cyst. Am J Clin Oncol.

[18] Hong KH, Moon WS, Chung GH (1999). Radiological appearance of primary branchial cleft cyst carcinoma. J Laryngol Otol.

[19] Joshi MJ, Provenzano MJ, Smith RJ (2009). The rare third branchial cleft cyst. AJNR Am J Neuroradiol.

